# Neuronal surface autoantibodies at cerebellar and brainstem synapses: mechanisms, phenotypes, and translational gaps

**DOI:** 10.3389/fimmu.2026.1962978

**Published:** 2026-10-07

**Authors:** Yan Li, Wenyuan Li, Zhaoben Zhang, Yuyuan Hu, Xinyu Ma, Zhijun Wang, Wei Zhang, Yanhong Wang

**Affiliations:** 1 Department of Neurology, Third Hospital of Shanxi Medical University, Shanxi Bethune Hospital, Shanxi Academy of Medical Sciences, Tongji Shanxi Hospital, Taiyuan, China; 2 Department of Neurosurgery, Third Hospital of Shanxi Medical University, Shanxi Bethune Hospital, Shanxi Academy of Medical Sciences, Tongji Shanxi Hospital, Taiyuan, China; 3 Division of Colorectal Surgery, Third Hospital of Shanxi Medical University, Shanxi Bethune Hospital, Shanxi Academy of Medical Sciences, Tongji Shanxi Hospital, Taiyuan, China; 4 Yuncheng Central Hospital, Shanxi Medical University, Yuncheng, China

**Keywords:** autoimmune encephalitis, brainstem, cerebellum, glycine receptor, mGluR1, neuronal surface autoantibodies, synaptic dysfunction

## Abstract

Pathogenic neuronal-surface autoantibodies can disrupt extracellular receptors, ion-channel complexes, and synaptic proteins, yet their regional neurological phenotypes differ substantially. This variability is particularly informative in the cerebellum and brainstem, where precisely timed excitatory and inhibitory signaling supports motor coordination, ocular-motor control, autonomic regulation, arousal, and respiration. In this Mini Review, we examine how antibody effector mechanisms, regional antigen distribution, and local synaptic physiology interact to shape cerebellar and brainstem manifestations of neuronal-surface-antibody disease. Autoimmunity against metabotropic glutamate receptor 1 (mGluR1) provides a model of cerebellar synaptic dysfunction because mGluR1 is strongly linked to Purkinje-cell signaling and synaptic plasticity, whereas glycine receptor (GlyR) autoimmunity illustrates how impaired inhibitory transmission can destabilize brainstem and spinal networks. NMDAR, AMPAR, GABA_A receptor, GABA_B receptor, LGI1, and CASPR2 autoimmunity provide mechanistic comparators showing that extracellular antigen accessibility alone does not determine neurological phenotype. Direct antibody-mediated synaptic dysfunction may be reversible, but persistent structural injury occurs in a subset of patients; current evidence does not determine whether such injury is attributable to sustained antibody exposure, accompanying T-cell inflammation, or both. Evidence from acute physiological experiments, individual follow-up cases, and heterogeneous cohorts comes from different settings and does not establish a uniform progression from synaptic dysfunction to atrophy.

## 1 Introduction

Neuronal-surface autoantibody disorders represent a mechanistically distinctive group of autoimmune neurological diseases because their target epitopes are accessible on receptors, ion-channel-associated complexes, and other proteins exposed at the neuronal membrane or synapse. In several well-characterized disorders, patient immunoglobulin G can directly alter the abundance, localization, molecular interactions, or function of its target rather than acting solely as a biomarker of an accompanying immune response (1, 2). Depending on the antigen and antibody properties, these effects include receptor cross-linking and internalization, functional blockade, disruption of extracellular protein–protein interactions, and changes in synaptic organization, ultimately modifying neuronal excitability, synaptic transmission, or plasticity (1, 3). Extracellular target accessibility is therefore important for direct antibody pathogenicity, but it does not by itself explain why neuronal-surface autoantibodies produce markedly different neurological phenotypes (1, 2).

The cerebellum and brainstem provide particularly informative settings in which to examine this problem because their functions depend on highly specialized synaptic signaling and precise control of neuronal output. Purkinje cells integrate mossy-fiber-driven parallel-fiber input and climbing-fiber signals and project to the deep cerebellar nuclei, which contribute to cerebellar outputs involved in coordination, adaptation, ocular-motor control, and predictive motor behavior (4, 5). Autoimmunity against metabotropic glutamate receptor 1 (mGluR1) illustrates how disruption of a synaptic protein closely linked to Purkinje-cell physiology can preferentially produce a cerebellar phenotype. mGluR1-dependent signaling contributes to parallel fiber–Purkinje-cell synaptic function and long-term depression, and anti-mGluR1-associated disease is characterized predominantly by acute or subacute cerebellar manifestations, including gait and limb ataxia, dysarthria, nystagmus, vertigo, and other ocular-motor abnormalities (6–8). In contrast, glycine receptors mediate fast inhibitory neurotransmission and are prominently expressed in the human brainstem and spinal cord (9). GlyR autoantibodies have been associated most strongly with progressive encephalomyelitis with rigidity and myoclonus and related stiff-person-spectrum phenotypes, in which rigidity, spasms, myoclonus, brainstem dysfunction, autonomic disturbance, and respiratory involvement may occur; experimental observations further support antibody-mediated GlyR internalization and degradation as one pathogenic mechanism (10, 11).

These contrasting examples suggest that neuronal-surface autoantibody disease is more informative when considered at the level of the targeted synapse than by extracellular antigen accessibility alone. Similar effector mechanisms can generate different regional phenotypes because their consequences depend on target distribution and the physiological role of the affected synapse (1–3).

We selected mGluR1 and GlyR as complementary anchors for cerebellar Purkinje-cell plasticity and fast brainstem–spinal inhibition, respectively (12, 13). The supporting experimental and clinical evidence differs between these targets (10, 12, 13). N-methyl-D-aspartate receptor (NMDAR), α-amino-3-hydroxy-5-methyl-4-isoxazolepropionic acid receptor (AMPAR), and γ-aminobutyric acid type A and B receptors (GABA_A and GABA_B receptors) provide receptor comparators. Leucine-rich glioma-inactivated 1 (LGI1) and contactin-associated protein-like 2 (CASPR2) provide synaptic-organizing protein comparators. Their use as comparators does not constitute a ranking by clinical importance. This focused narrative Mini Review prioritizes original studies connecting antibody action with regional expression, circuit physiology, and clinical phenotype, supplemented by clinical cohorts and reviews. We also consider limited or conflicting evidence, without claiming a systematic or exhaustive search. We examine how antibody mechanism, regional antigen distribution, and synaptic physiology jointly shape phenotype and reversibility.

## 2 Synaptic effector mechanisms of neuronal-surface autoantibodies

Neuronal-surface autoantibodies do not perturb synapses through a single molecular mechanism. Antibody binding can reduce receptor availability, perturb receptor function without detectable receptor loss, or disrupt extracellular organizing interactions (14–17). Effects vary with antigen organization, antibody properties, and epitope specificity. These mechanisms are not mutually exclusive, and their physiological consequences depend on the function of the affected synaptic system. The following examples compare how these mechanisms affect excitatory receptors, inhibitory receptors, and extracellular synaptic-organizing proteins. Antibody-induced loss of surface receptors is best established in anti-NMDAR encephalitis. Patient IgG cross-links GluN1-containing NMDARs, producing a selective, titer-dependent reduction in surface and synaptic receptors with corresponding loss of NMDAR-mediated currents; receptor expression can recover after antibody withdrawal in experimental systems (15). AMPAR antibodies similarly reduce the number and synaptic localization of GluA1/GluA2-containing receptors, with recovery after antibody withdrawal (18). Receptor depletion also affects inhibitory synapses: patient sera reduced surface and synaptic GABA_A receptors and miniature inhibitory postsynaptic currents in cultured neurons (19). Thus, receptor depletion represents a recurrent mechanism of neuronal-surface autoimmunity, but its circuit-level consequence depends on whether the depleted receptor normally supports excitation, inhibition, or synaptic plasticity.

Direct antibody effects can also occur without substantial receptor internalization. GABA_B receptor antibodies illustrate a distinct mechanism in which patient IgG alters receptor-dependent neuronal and network activity without measurable loss of surface GABA_B receptors, demonstrating that functional pathogenicity does not require receptor depletion (14).

Autoantibodies directed against extracellular synaptic-organizing proteins illustrate a mechanistically different form of dysfunction. LGI1 antibodies can interfere with LGI1 binding to ADAM22/ADAM23 and thereby disturb a trans-synaptic complex involved in the regulation of excitability and synaptic transmission (20). LGI1 and CASPR2 autoantibodies illustrate disruption of extracellular organizing interactions rather than simple receptor depletion: LGI1 monoclonal antibodies can either block LGI1–ADAM22/23 docking or promote internalization of the complex, whereas CASPR2 antibodies inhibit CASPR2–contactin-2 binding without substantial CASPR2 internalization (16, 17).

GABA_A receptor and GlyR autoimmunity illustrate weakened inhibition through receptor depletion or direct functional interference (10, 13, 19). GABA_B receptor antibodies instead demonstrate altered network activity without detectable receptor loss, emphasizing that inhibitory targets need not produce identical physiological effects (14). Antigen accessibility therefore permits direct antibody–target interaction but does not specify its physiological consequence, which depends on both the effector mechanism and the function of the affected synapse (1). Current mechanistic evidence is strongest at the receptor, synaptic, and circuit levels, where neuronal-surface autoantibodies have been shown to alter receptor trafficking or extracellular protein interactions and to modify synaptic currents, neuronal firing, synaptic plasticity, and network activity (12, 13, 15, 16). Metabolic abnormalities have also been observed at the systems level, including antibody-associated regional hyper- and hypometabolism on FDG-PET and altered glutamatergic metabolite profiles in anti-NMDAR encephalitis; however, these observations do not establish a direct metabolic effect of antibody binding on individual neurons (21–24). Whether neuronal-surface autoantibodies induce reproducible, antigen-specific changes in neuronal bioenergetics or broader metabolic programs therefore remains insufficiently defined and represents an important mechanistic gap (21, 25).

GABA_A receptor autoimmunity illustrates both receptor depletion in patient-serum-treated neurons and direct functional antagonism by patient-derived monoclonal antibodies (19, 26). GlyR antibodies likewise affect receptor trafficking and can rapidly suppress glycinergic currents (10, 13). The brief GABA_A receptor discussion reflects this review’s focus on cerebellar plasticity and brainstem–spinal inhibition, rather than a weaker overall evidence base. Representative experimental findings and study-specific limitations are summarized in **Table 1**.

**Table 1:** Representative experimental evidence for direct pathogenic effects of neuronal-surface autoantibodies.

Target	Representative experimental model/material	Direct antibody effect	Functional consequence	Key limitation
NMDAR	Cultured rat hippocampal neurons exposed to patient CSF/IgG	Antibody-mediated cross-linking and internalization with selective, titer-dependent loss of surface and synaptic NMDARs	Reduced NMDAR-mediated synaptic currents; receptor loss reversible after antibody withdrawal	Experimental neuronal model does not reproduce the complete human syndrome
AMPAR	Live rat hippocampal neurons exposed to patient antibodies	Reduced number and synaptic localization of GluA1/GluA2-containing AMPARs	Reduced synaptic AMPAR availability; effects reversed after antibody removal	Initial mechanistic evidence derived from a small clinical cohort and neuronal cultures
GABA_A receptor	Cultured rat hippocampal neurons exposed to sera from antibody-positive patients	Reduced surface and synaptic GABA_A receptors	Reduced miniature inhibitory postsynaptic current amplitude and frequency	Patient-derived material was limited; both reported patients had additional neuronal autoantibodies
GABA_B receptor	Primary hippocampal cultures and acute medial entorhinal cortex slices exposed to patient IgG	No measurable reduction in surface GABA_B receptor expression despite antibody exposure	Reduced network UP-state duration and pyramidal-cell spike rate	Functional study used IgG from a single GABA_B receptor-antibody-positive patient
LGI1	HEK293T cells, live hippocampal neurons, and rodent intrahippocampal injection using patient-derived monoclonal antibodies	LRR-directed mAbs promoted LGI1–ADAM22/23 complex internalization; EPTP-directed mAbs inhibited LGI1 docking to ADAM22/23	Impaired long-term potentiation; selected mAbs produced memory impairment *in vivo*	Monoclonal antibodies were generated from two patients and may not represent the full polyclonal response
CASPR2	Cell-based interaction assays and neuronal preparations exposed to patient antibodies	Inhibition of CASPR2–contactin-2 interaction without substantial CASPR2 internalization	Disruption of CASPR2-dependent extracellular molecular organization	Experimental study did not reproduce the complete clinical phenotype *in vivo*
mGluR1	Patient IgG applied to mouse cerebellar slices, mouse flocculus *in vivo*, and cultured Purkinje cells; separate ocular-motor assessment in affected patients	Acute disruption of mGluR1-dependent Purkinje-cell function	Reduced Purkinje-cell basal firing and blocked LTD in experimental preparations; impaired compensatory eye movements in mice; impaired saccadic adaptation observed in patients	Evidence derives from rare predominantly paraneoplastic cases; effects may not be identical across patients
GlyR	HEK/cellular trafficking assays, cultured spinal motor neurons, brainstem slices, and passive-transfer models using patient IgG/sera	Receptor internalization and lysosomal targeting; rapid direct functional inhibition; presynaptic as well as postsynaptic effects	Reduced glycinergic inhibitory transmission and experimentally reproduced motor/behavioral dysfunction	Experimental preparations and exposure conditions differ across studies and no single model reproduces the full human PERM/SPS phenotype

Representative studies were selected to illustrate distinct experimentally demonstrated antibody effector mechanisms rather than to provide a systematic ranking of pathogenicity or evidence strength. Experimental systems, antibody preparations, exposure conditions, and outcome measures differ substantially across studies and should not be interpreted as directly comparable. AMPAR, α-amino-3-hydroxy-5-methyl-4-isoxazolepropionic acid receptor; CASPR2, contactin-associated protein-like 2; CSF, cerebrospinal fluid; EPTP, epitempin repeat domain; GABA_A receptor, γ-aminobutyric acid type A receptor; GABA_B receptor, γ-aminobutyric acid type B receptor; GlyR, glycine receptor; IgG, immunoglobulin G; LGI1, leucine-rich glioma-inactivated 1; LRR, leucine-rich repeat domain; LTD, long-term depression; mAb, monoclonal antibody; mGluR1, metabotropic glutamate receptor 1; NMDAR, N-methyl-D-aspartate receptor; PERM, progressive encephalomyelitis with rigidity and myoclonus; SPS, stiff-person syndrome.

## 3 Region-specific synaptic dysfunction in the cerebellum and brainstem

### 3.1 mGluR1 autoimmunity: cerebellar synaptic plasticity and motor adaptation

mGluR1 autoimmunity provides an unusually direct example of how regional antigen distribution and synaptic physiology can shape the phenotype of neuronal-surface autoantibody disease. mGluR1 is strongly expressed in cerebellar Purkinje cells and is concentrated postsynaptically and perisynaptically on Purkinje-cell dendritic spines receiving parallel-fiber input (27–29). At the parallel fiber–Purkinje-cell synapse, mGluR1 activation generates slower postsynaptic signaling coupled to intracellular calcium mobilization and participates in synaptic plasticity, including mechanisms required for induction and regulation of long-term depression (LTD) (30–32). This anatomical and physiological concentration provides a plausible basis for the striking cerebellar predominance of anti-mGluR1-associated disease rather than merely identifying the cerebellum as another region in which the antigen happens to be expressed.

Direct experimental evidence supports a pathogenic effect of mGluR1 autoantibodies on this synaptic system. Experiments using patient-derived IgG showed acute suppression of Purkinje-cell basal firing, impaired compensatory eye movements after cerebellar application *in vivo*, and blockade of LTD induction in cultured Purkinje cells; affected patients also demonstrated impaired saccadic adaptation (12). These experimental and clinical findings support anti-mGluR1 disease as a model of cerebellar synaptopathy (12). Effects across patients and downstream cerebellar output pathways require further characterization (5, 6, 12). They therefore support anti-mGluR1 disease as a useful model of antibody-mediated cerebellar synaptopathy, although the precise antibody effects may not be identical across patients or epitopes (6, 12). This mechanism-to-circuit link is summarized in **Supplementary Figure 1**.

The clinical phenotype is consistent with this mechanistic localization. Contemporary syntheses confirm predominant cerebellar involvement, with gait or limb ataxia, dysarthria, nystagmus, vertigo, tremor, and other ocular-motor abnormalities among the most frequent manifestations, while behavioral changes, cognitive impairment, hallucinations, and seizures have also been reported in a minority of cases (7, 8). The close correspondence among regional receptor enrichment, experimentally demonstrated disruption of Purkinje-cell physiology, and the clinical phenotype makes mGluR1 autoimmunity particularly informative for the target–synapse–circuit–phenotype framework proposed here (8, 12).

### 3.2 GlyR autoimmunity: brainstem–spinal inhibitory transmission

GlyRs are chloride-permeable ligand-gated receptors mediating fast inhibition, with prominent expression in the brainstem and spinal cord (9). Antibody-mediated disruption of glycinergic transmission provides a mechanistic link to motor hyperexcitability and brainstem–spinal dysfunction (13).

Experimental studies support multiple antibody-mediated mechanisms underlying GlyR dysfunction. GlyR antibodies can induce receptor internalization and lysosomal targeting (10). Rapid suppression of glycinergic currents, including by monovalent Fab fragments, additionally supports direct functional inhibition (13). Recent brainstem-slice experiments further extend this model to presynaptic GlyRs, where autoantibody exposure reduced the frequency of spontaneous and miniature inhibitory postsynaptic potentials (33).

The clinical phenotype of GlyR antibody-associated disease reflects the central role of glycinergic inhibition in motor and brainstem–spinal circuits. GlyR antibodies are most strongly associated with progressive encephalomyelitis with rigidity and myoclonus (PERM) and related stiff-person-spectrum disorders, characterized by rigidity, painful spasms, stimulus-sensitive myoclonus, exaggerated startle responses, and frequently brainstem or autonomic manifestations (10, 13). Because glycinergic inhibition contributes to spinal motor control and brainstem respiratory patterning, disruption of GlyR-mediated transmission provides a plausible mechanistic link to motor hyperexcitability and respiratory involvement in affected patients (9, 13, 34). GlyR-antibody-associated disease is not restricted to classical PERM or stiff-person-spectrum disorders; broader reported phenotypes include seizures, cerebellar ataxia, movement disorders, encephalitic syndromes, and generalized weakness involving respiratory muscles (11).

Together, mGluR1 and GlyR autoimmunity illustrate how disruption of cerebellar plasticity and brainstem–spinal inhibition can produce contrasting regional phenotypes (12, 13).

## 4 From antigen accessibility to regional phenotype: context and reversibility

Extracellular accessibility and even similar antibody effector mechanisms do not determine phenotype. NMDAR and AMPAR antibodies both reduce synaptic receptor availability, yet NMDAR autoimmunity typically produces a distributed encephalitic syndrome whereas AMPAR autoimmunity is predominantly associated with limbic encephalitis (15, 18, 35). Thus, similar receptor-directed mechanisms can generate different regional phenotypes.

A second layer of heterogeneity arises from the molecular organization of the targeted synapse. LGI1 and CASPR2 antibodies both recognize proteins associated with extracellular synaptic or axonal organization, but their pathogenic effects and clinical distributions are not interchangeable. LGI1 autoantibodies can disrupt LGI1–ADAM22/ADAM23 interactions or induce internalization of LGI1-containing complexes, with different monoclonal antibodies producing distinct effects according to the recognized domain (16). Antibody properties may also associate with clinical heterogeneity within LGI1 encephalitis itself, including differences between limbic encephalitis and faciobrachial dystonic seizure phenotypes (36). CASPR2 autoantibodies, in contrast, can inhibit CASPR2–contactin-2 interactions without substantial CASPR2 internalization, and CASPR2-associated disease spans limbic encephalitis, peripheral nerve hyperexcitability, Morvan syndrome, dysautonomia, pain, sleep disturbance, and occasional cerebellar manifestations (17, 37). These observations indicate that extracellular localization defines the opportunity for direct antibody action but does not specify its physiological consequence.

We therefore propose that regional phenotype should be interpreted at the intersection of three variables: antibody effector mechanism, regional antigen distribution, and synaptic physiological function. Antibody mechanism determines how the molecular target is perturbed; antigen distribution constrains which neural systems are directly exposed to that perturbation; and synaptic physiology determines what information-processing function is lost when the target is disrupted, including how local microcircuit dysfunction may be transmitted through cerebellar or brainstem output pathways (5, 38). This framework is explanatory rather than predictive and does not imply that these variables act independently or are sufficient to account for all clinical heterogeneity. In anti-NMDAR neuron–microglia co-cultures, microglia removed antibody-bound receptors and associated postsynaptic proteins (39). This finding does not establish analogous mechanisms in anti-mGluR1 or anti-GlyR disease *in vivo*. Nevertheless, organizing neuronal-surface autoimmunity around the targeted synapse provides a mechanistically sharper explanation for regional neurological phenotypes than extracellular antigen accessibility alone. This conceptual relationship is summarized in **Figure 1**, which integrates synaptic mechanisms with a simplified cerebellar macro-circuit linking local cerebellar dysfunction to deep cerebellar nuclear and downstream brainstem/thalamic-cortical pathways (5).

**Figure 1:**
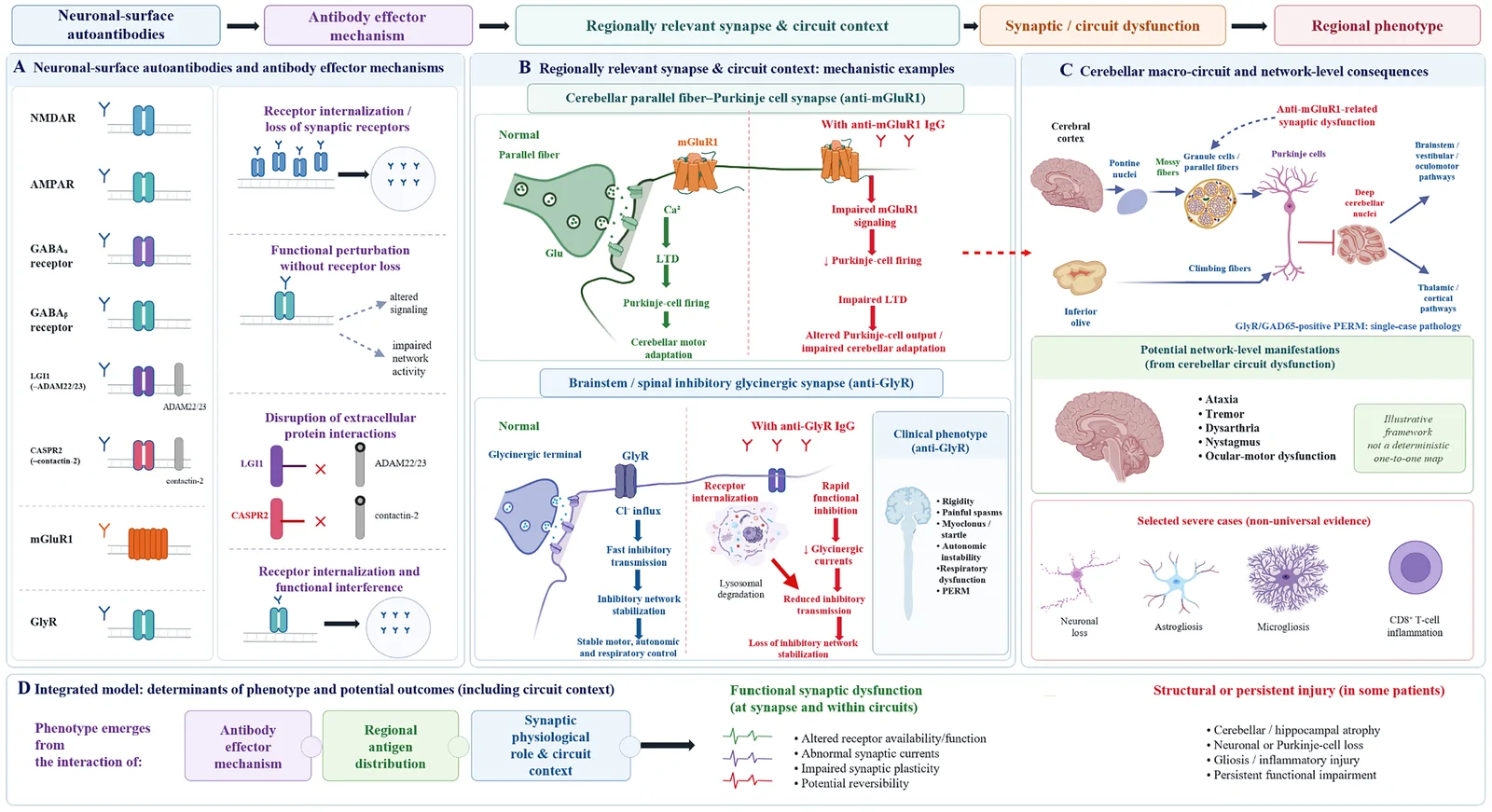
Synapse-centered framework linking neuronal-surface autoantibody mechanisms to regional neurological phenotypes across synaptic and circuit levels. **(A)** Representative neuronal-surface autoantibodies perturb synaptic function through distinct effector mechanisms, including receptor cross-linking and internalization, functional interference without substantial receptor loss, and disruption of extracellular protein interactions. **(B)** Anti-mGluR1 and anti-GlyR autoimmunity provide complementary mechanistic and microcircuit anchors. Patient IgG reduced Purkinje-cell basal firing, blocked LTD in culture, and impaired compensatory eye movements in mice. Impaired saccadic adaptation was observed separately in affected patients. At glycinergic brainstem/spinal synapses, anti-GlyR antibodies can promote receptor internalization and lysosomal degradation and can also rapidly inhibit receptor function, thereby reducing inhibitory transmission and destabilizing motor, autonomic, and respiratory networks. **(C)** A simplified cerebellar macro-circuit illustrates how local synaptic dysfunction may influence larger-scale network output. Cerebral cortical signals are relayed through the pontine nuclei to mossy fibers, granule cells, and parallel fibers, whereas inferior-olivary climbing fibers provide a second major input to Purkinje cells. Purkinje cells provide inhibitory output to the deep cerebellar nuclei, which in turn project to brainstem/vestibular/oculomotor pathways and to thalamic/cortical circuits. Within this framework, anti-mGluR1-related synaptic dysfunction may therefore contribute to network-level manifestations such as ataxia, tremor, dysarthria, nystagmus, and ocular-motor dysfunction. These relationships are illustrative rather than a deterministic one-to-one mapping between a specific antibody and a particular clinical manifestation. The lower inset illustrates neuronal loss, astrogliosis, microgliosis, and CD8^+^ T-cell inflammation reported in a fatal PERM case with coexisting GlyR and GAD65 antibodies. This single-case pathology cannot be generalized to isolated GlyR autoimmunity or anti-mGluR1 disease. **(D)** The integrated model proposes that regional phenotype emerges from the interaction among antibody effector mechanism, regional antigen distribution, and the physiological role and downstream circuit context of the targeted synapse. Potentially reversible synaptic dysfunction and persistent structural injury are distinct outcomes that may coexist in individual patients. Their juxtaposition does not establish a temporal progression or a shared causal mechanism. Reported structural findings include cerebellar or hippocampal atrophy, neuronal or Purkinje-cell loss, and gliosis or inflammatory injury. The model is explanatory rather than predictive and does not assign structural injury to sustained antibody exposure, T-cell inflammation, or their combination.

Non-motor and psychiatric features have been reported in some neuronal-surface-autoantibody disorders, including anti-mGluR1, NMDAR, and LGI1 encephalitis (40, 41). These observations do not establish a cerebellar cause or a pathogenic role for serum neuronal antibodies in primary psychiatric disorders when CSF confirmation, syndrome concordance, or functional evidence is lacking (42, 43).

In cultured neurons, patient anti-NMDAR antibodies caused a selective reduction in surface and synaptic NMDARs that recovered after antibody withdrawal, without immediate neuronal loss (15). This demonstrates reversibility of a defined receptor-level effect under experimental conditions. Separately, two selected patients with severe anti-NMDAR encephalitis showed improvement of frontotemporal atrophy and hypoperfusion on imaging 5–7 years later, but these cases do not establish a uniform clinical trajectory (44).

Structural sequelae have also been reported in neuronal-surface-autoantibody disorders, including progressive cerebellar atrophy in anti-NMDAR encephalitis and hippocampal injury in anti-LGI1 encephalitis (45–47). These observations show that structural injury can occur in such disorders, but they do not establish the mechanism of cerebellar atrophy in anti-mGluR1 disease.

Anti-mGluR1 findings come from distinct experimental, pathological, and longitudinal observations and should not be treated as a single disease sequence. Patient IgG acutely altered Purkinje-cell firing, compensatory eye movements, and LTD; the same clinico-experimental study reported marked Purkinje-cell loss in one paraneoplastic postmortem case (12). A treated, non-paraneoplastic case developed progressive cerebellar atrophy during five-year follow-up, despite nearly stable ataxia and persistently detectable serum antibodies (48). This observation excludes neither antibody-mediated injury nor accompanying cellular inflammation. Seven of 32 patients with longitudinal MRI developed cerebellar atrophy after 10–48 months (40). These observations came from published cases; the 11 new cases had no follow-up MRI at the study cutoff. Malignancy occurred in 9 of 53 pooled cases, but that overall count does not identify the mechanism of atrophy.

A fatal PERM case with coexisting GlyR and GAD65 antibodies showed neuronal loss, astrogliosis, microgliosis, and CD8+ T-cell inflammation (49). Because this is a single dual-antibody case, the pathology cannot be attributed specifically to GlyR antibodies or generalized to isolated GlyR autoimmunity.

Antibody-mediated synaptic dysfunction can be reversible, while structural injury occurs in selected patients (12, 15, 40, 48). Current evidence does not establish a uniform progression between these outcomes or distinguish sustained antibody effects from accompanying T-cell inflammation. Tumor status, onset-to-treatment interval, serial imaging, and available pathological findings should therefore be reported when interpreting apparent reversibility. **Supplementary Table S1** and **Supplementary Figure 2** summarize these distinct or coexisting possibilities without assigning structural injury to a specific immune mechanism.

## 5 Discussion: translational gaps and future directions

Three translational priorities and their target-specific considerations are summarized in **Supplementary Table S2**. First, antibody detection must be distinguished from pathogenicity. A positive cell-based assay establishes antigen recognition but not causal relevance, and interpretation should incorporate phenotype concordance, confirmatory testing, and antigen-specific guidance on the most informative sample type (50, 51). In anti-NMDAR encephalitis, for example, CSF testing is more sensitive than serum testing (52, 53). For incompletely characterized antibodies, neuronal functional assays, patient-derived monoclonal antibodies, or passive-transfer experiments can provide stronger evidence of causality (16, 54).

Second, a disease-specific gap is validation of ocular-motor endpoints for longitudinal assessment in anti-mGluR1 disease. Patient IgG impaired compensatory eye movements experimentally, whereas affected patients exhibited impaired saccadic adaptation (12). Prospective studies should prespecify eye-movement tasks and quantitative metrics, include healthy and cerebellar disease controls, and repeat the protocol before and after treatment. Measurements should be paired with standardized ataxia scores and MRI, with blinded assessment, test–retest reliability, and multicenter replication. Clinically meaningful change must be established, considering small cohorts, heterogeneous disease courses, and nonspecific ocular-motor abnormalities. For GlyR disease, target-relevant physiology should instead accompany systematic motor, autonomic, and respiratory assessment (13). **Supplementary Figure 1** outlines this proposed validation pathway for anti-mGluR1 disease.

Third, functional recovery and structural injury require longitudinal assessment that links measurements obtained within the same visit (**Supplementary Figure 2**). Antibody measurements, target-relevant physiology, standardized clinical outcomes, and structural MRI should be interpreted together across visits. CSF sampling should be determined by clinical indication and feasibility rather than mandated at every visit. Studies should record tumor status, symptom duration, treatment delay, and immunotherapy exposure, while maintaining consistent physiological protocols and MRI sequences (12, 40, 48). Initial studies should establish feasibility and reproducibility before attempting individual-outcome prediction. The illustrated trajectories are hypotheses for prospective testing, rather than patient data or a validated prediction model.

This synapse-centered framework relates antibody mechanism, antigen distribution, and synaptic physiology to regional phenotype. Its clinical value depends on validating target-relevant physiological endpoints and interpreting functional recovery alongside structural findings (12, 15, 40, 48). It remains an explanatory framework rather than a validated tool for predicting individual outcomes.
