# Supplementary material for: Neuronal surface autoantibodies at cerebellar and brainstem synapses: mechanisms, phenotypes, and translational gaps
Source: Front Immunol. 2026 Oct 7;17:1962978. doi: 10.3389/fimmu.2026.1962978 (PMC13684871; doi:10.3389/fimmu.2026.1962978)
Supplement: Supplementary file 1 [file SupplementaryFile1.docx]

**Supplementary Table S1. Evidence supporting and challenging reversibility in neuronal-surface-autoantibody disorders**

| **Disorder/target** | **Study design and sample** | **Evidence compatible with reversibility** | **Evidence of structural or persistent injury** | **Outcome/time context** | **Interpretation for this Review** | **Key limitation** | **Anti-mGluR1 case/cohort context: tumor; treatment delay; atrophy timing; pathology** |
| --- | --- | --- | --- | --- | --- | --- | --- |
| Anti-NMDAR | *In vitro* neuronal study | Antibody-induced surface/synaptic NMDAR loss reversed after antibody removal | Immediate neuronal loss not demonstrated in the experimental system | Experimental exposure/recovery | Strong basis for **potentially reversible receptor dysfunction** | Does not establish reversibility of whole human disease | Not available |
| Anti-NMDAR | Long-term observational report; 2 women with severe disease | Marked frontotemporal atrophy and hypoperfusion substantially improved | Not reported as persistent structural loss | MRI/SPECT reassessed 5–7 years later; cognition improved gradually | Severe radiological abnormalities can be reversible in selected patients | n=2; prolonged critical illness/anesthetic exposure | Not available |
| Anti-NMDAR | Retrospective longitudinal imaging study; n=15 | Diffuse cerebral atrophy could reverse | Progressive cerebellar atrophy was irreversible and associated with poor outcome | Serial MRI/long-term outcome | Reversibility is **region- and trajectory-dependent**, not guaranteed by antigen topology | Small, single-center retrospective cohort | Not available |
| Anti-LGI1 | Nationwide cohort; n=38 | Substantial response to immunotherapy in 80% | Initial hippocampal T2 abnormalities regularly evolved to mesial temporal sclerosis (44%) | Long-term clinical/MRI follow-up | Clinical responsiveness can coexist with lasting structural sequelae | Observational cohort; no direct antibody-to-atrophy causality | Not available |
| Anti-LGI1 | Cross-sectional MRI/cognitive study; 30 patients + 27 controls | Not designed to assess acute reversibility | Pronounced hippocampal/subfield atrophy and microstructural injury correlated with persistent memory deficits | Post-acute structural and cognitive assessment | Demonstrates clinically meaningful structural damage after surface-antibody encephalitis | Cross-sectional; cannot define individual temporal sequence | Not available |
| Anti-LGI1 | Post-acute imaging study; 34 patients + 34 controls | Increased hippocampal–mOFC connectivity was associated with better memory performance | Bilateral hippocampal atrophy correlated with poorer cognition | Structural MRI + resting-state fMRI, 2026 | Structural injury and functional compensation may coexist | Association does not prove that enhanced connectivity causes recovery | Not available |
| Anti-mGluR1 | Clinico-experimental/pathological study | Acute antibody effects on Purkinje firing and LTD demonstrate a functional component | One postmortem paraneoplastic case showed approximately two-thirds Purkinje-cell reduction vs controls | Acute experimental effects + postmortem pathology | Functional synaptopathy and neuronal loss are not mutually exclusive | Pathological finding from one paraneoplastic patient | Tumor status: Paraneoplastic (postmortem case; Hodgkin disease).  Onset to immunotherapy: Not reported.  Atrophy detection: Not reported; the finding was postmortem Purkinje-cell loss.  Pathology: Yes; one autopsy with approximately two-thirds fewer Purkinje cells versus three controls |
| Anti-mGluR1 | Single-patient 5-year follow-up | Ataxia remained approximately stable with repeated immunotherapy | Cerebellar atrophy gradually progressed | 5-year longitudinal observation | Clinical stabilization does not necessarily imply structural stabilization | Single case | Tumor status: Non-paraneoplastic.  Onset to immunotherapy: Approximately 2 months, as tabulated in a later case-level review; repeated IVIG and oral immunosuppression during follow-up.  Atrophy detection: MRI at 57 months after onset in the later case-level review; gradual progression during 5 years.  Pathology: Not reported |
| Anti-mGluR1 | Integrated cohort; 53 patients | 65.9% of patients with available outcome data improved clinically | 7/32 longitudinally assessed patients developed cerebellar atrophy; initially normal MRI could precede atrophy | Follow-up atrophy latency 10–48 months in reported cases | Normal initial MRI cannot establish purely functional disease | Heterogeneous pooled literature + single-center cases; non-prospective review | Tumor status: Malignancy in 9/53 overall; status of the 7 atrophy cases not available as an adequately stratified subset.  Onset to immunotherapy: Not available for the 7 atrophy cases as a subset.  Atrophy detection: 7/32 longitudinal MRI patients; initial MRI normal, with reported latency of 10–48 months.  Pathology: Not reported for the atrophy subgroup. |
| GlyR + GAD65 PERM | Single fatal clinico-pathological case | Not demonstrated; disease refractory despite extensive immunotherapy | Neuronal loss, astrogliosis, microgliosis, CD8+ T-cell inflammation and pallido-nigro-dentato-bulbar-spinal degeneration | Death 10 months after onset | Shows that severe PERM can contain substantial inflammatory/degenerative pathology | **Dual GlyR/GAD65 positivity; single case; injury cannot be assigned specifically to GlyR** | **Not available** |

**Note:** The studies summarized here differ in disease subtype, sample size, severity, treatment exposure, imaging methodology, pathological sampling, and duration of follow-up. The table illustrates the coexistence of potentially reversible functional abnormalities and persistent structural injury; it does not compare their frequencies across antibody-defined diseases. For the anti-mGluR1 rows, tumor status, onset-to-immunotherapy timing, time of atrophy detection, and pathology are reported only when available for the specific case or cohort. The 53-patient integrated cohort reported malignancy in 9/53 patients and cerebellar atrophy in 7/32 patients with longitudinal MRI, but did not adequately stratify the atrophy cases by tumor status, treatment delay, or pathology. A separate non-paraneoplastic patient had persistently detectable serum antibodies and progressive atrophy during five years of repeated immunotherapy, with ataxia remaining nearly stable; this single case does not establish antibody-mediated causation or exclude T-cell involvement. For that patient, the approximately 2-month interval to treatment and MRI detection of atrophy at 57 months after onset come from a later published case-level table. The postmortem Purkinje-cell loss was observed in one paraneoplastic patient and should not be merged with the separate longitudinal cases as a single disease trajectory. The integrated cohort includes previously published cases, so the anti-mGluR1 rows are not independent patient groups. The GlyR and GAD65 dual-antibody case remains non-specific for GlyR attribution. ‘Not available’ in the added context column for other targets means no additional case-level extraction was undertaken for this focused revision. CSF, cerebrospinal fluid; DCA, diffuse cerebral atrophy; fMRI, functional magnetic resonance imaging; GAD65, 65-kDa glutamic acid decarboxylase; GlyR, glycine receptor; LGI1, leucine-rich glioma-inactivated 1; LTD, long-term depression; mGluR1, metabotropic glutamate receptor 1; mOFC, medial orbitofrontal cortex; MRI, magnetic resonance imaging; MTS, mesial temporal sclerosis; NMDAR, N-methyl-D-aspartate receptor; PERM, progressive encephalomyelitis with rigidity and myoclonus; SPECT, single-photon emission computed tomography..

**Supplementary Table S2. Evidence and assessment domains relevant to mechanistic phenotyping of neuronal-surface autoantibody disorders**

| **Translational question** | **Current interpretive problem** | **Evidence/assessment needed** | **Representative supporting evidence** | **What the evidence can establish** | **What it cannot establish alone** |
| --- | --- | --- | --- | --- | --- |
| **Is the detected antibody clinically relevant?** | A positive neuronal-antibody result may be incidental or discordant with the syndrome | Phenotype concordance; appropriate sample; confirmatory testing; exclusion of alternative diagnoses | In one referral cohort, many antibody-positive results were ultimately judged clinically irrelevant; consensus guidance emphasizes contextual interpretation | Strengthens clinical relevance of antibody positivity | Does not by itself demonstrate direct pathogenicity |
| **Does the assay detect the biologically relevant surface epitope?** | Fixation or assay configuration can alter accessibility of conformational extracellular epitopes | Antigen-specific CBA supplemented, when appropriate, by live-neuron/tissue-based confirmation | Diagnostic-method literature describes complementary roles and limitations of live neurons, tissue assays and CBAs | Confirms reproducible binding to an appropriately presented neuronal-surface antigen | Binding alone cannot prove that antibodies alter neuronal function |
| **Which biological compartment is most informative?** | Serum and CSF sensitivity/specificity differ by antigen | Paired serum/CSF interpreted using antigen-specific evidence | In anti-NMDAR encephalitis, CSF sensitivity was 100% vs 85.6% for serum in a 250-patient study; a Dutch cohort reported 99% vs 68% | Defines sample-specific diagnostic performance for **anti-NMDAR** testing | Cannot be generalized automatically to every neuronal-surface antibody |
| **Is the antibody functionally pathogenic?** | Antigen recognition does not establish a causal effect | Patient IgG/mAb exposure, receptor-trafficking assays, electrophysiology and—where feasible—passive transfer | LGI1 patient-derived mAbs produced domain-specific molecular effects; GlyR antibody passive transfer caused abnormal escape behavior in zebrafish | Moves evidence from association toward causal molecular/physiological action | Experimental phenotype may reproduce only part of the human syndrome |
| **What physiological function is disrupted?** | Antibody identity alone does not specify the functional deficit in an individual patient | Target-relevant physiological phenotyping linked to experimental antibody effects | mGluR1 IgG altered Purkinje firing/LTD and cerebellar adaptation; GlyR antibodies impaired inhibitory currents, with newer evidence for presynaptic involvement | Connects molecular target to measurable circuit/synaptic function | Does not yet provide a validated individual-patient prediction algorithm |
| **Is dysfunction predominantly functional or accompanied by structural injury?** | Normal early imaging or clinical improvement may falsely imply absence of tissue injury | Serial structural imaging integrated with repeated quantitative clinical/physiological assessment | Longitudinal NMDAR, mGluR1 and LGI1 studies demonstrate divergent structural trajectories and clinically relevant atrophy | Identifies evolving structural injury and clinicoradiological dissociation | A single MRI or treatment response cannot establish reversibility or irreversibility |

**Note:** The proposed framework represents a synthesis of current mechanistic and clinical evidence and is not a validated diagnostic or treatment algorithm. Assay choice and interpretation of serum and CSF must remain antigen specific. Functional assays and passive-transfer models strengthen evidence for pathogenicity but do not necessarily reproduce the complete human disease phenotype. Longitudinal assessment should integrate clinical phenotype, target-relevant physiology, and structural measures rather than infer reversibility from antibody classification or a single imaging time point. CBA, cell-based assay; CSF, cerebrospinal fluid; GlyR, glycine receptor; IgG, immunoglobulin G; LGI1, leucine-rich glioma-inactivated 1; mAb, monoclonal antibody; mGluR1, metabotropic glutamate receptor 1; MRI, magnetic resonance imaging; NMDAR, N-methyl-D-aspartate receptor.
