# Supplementary material for: Neuronal surface autoantibodies at cerebellar and brainstem synapses: mechanisms, phenotypes, and translational gaps
Source: Front Immunol. 2026 Oct 7;17:1962978. doi: 10.3389/fimmu.2026.1962978 (PMC13684871; doi:10.3389/fimmu.2026.1962978)
Supplement: Supplementary file 2 [file Table1.docx]

Supplementary Figure Legends

**Supplementary Figure 1.** Mechanistic rationale and prospective validation of ocular-motor measurements in anti-mGluR1 disease. **(A)** Simplified circuitry links parallel- and climbing-fiber inputs to Purkinje-cell output and downstream nuclei involved in ocular-motor control. **(B)** Canonical mGluR1-linked signaling supporting parallel fiber–Purkinje-cell LTD is contrasted with antibody-associated impairment of plasticity. The signaling cascade and AMPAR removal are schematic; direct antibody blockade of every depicted intermediate is not established. Coesmans et al. reported reduced Purkinje-cell firing, impaired compensatory eye movements in experimental animals, blocked LTD in cultured Purkinje cells, and impaired saccadic adaptation in patients(1). **(C)** Vestibular input and compensatory eye movements illustrate the rationale for ocular-motor measurements. Adaptation slope, VOR gain, latency, and inter-trial variability are candidate features; the traces are illustrative, not patient data. These features do not constitute a validated disease-specific biomarker for routine clinical use. **(D)** Proposed validation compares anti-mGluR1 patients with cerebellar disease controls and healthy controls using the same eye-tracking task at baseline, after treatment, and during follow-up. Priorities are standardization, discrimination from disease controls, multicenter reliability, and responsiveness to clinical change, with each readout linked to clinical severity and serial MRI. AMPAR, α-amino-3-hydroxy-5-methyl-4-isoxazolepropionic acid receptor; Gq, Gq-family G protein; IgG, immunoglobulin G; LTD, long-term depression; mGluR1, metabotropic glutamate receptor 1; MRI, magnetic resonance imaging; PKC, protein kinase C; PLCβ, phospholipase Cβ; VOR, vestibulo-ocular reflex.

**Supplementary Figure 2.** Longitudinal assessment of synaptic recovery and persistent injury. **(A)** A generic receptor-trafficking schematic illustrates potentially reversible synaptic dysfunction, alongside a separate depiction of Purkinje-cell degeneration and gliosis in selected cases. Teal structures denote membrane receptors, rose Y-shaped structures denote autoantibodies, and small dots in the synaptic cleft denote neurotransmitter molecules. These are distinct or potentially coexisting possibilities, not an established sequence. Mechanisms vary by target; the schematic does not imply identical receptor trafficking in mGluR1 and GlyR disease or determine the contributions of sustained antibody exposure and accompanying inflammation. **(B)** At each illustrative visit, antibody measurements, target-relevant physiology, structural MRI, and clinical outcomes are collected in parallel. Eye-movement tasks are candidate endpoints for mGluR1 disease; GlyR disease requires relevant physiology alongside motor, autonomic, and respiratory assessment. Sample selection follows antigen-specific guidance and feasibility; CSF sampling is not required at every visit. Tumor status, treatment delay, and immunotherapy exposure inform interpretation. **(C)** Conceptual trajectories illustrate recovery, divergence, and persistence of functional and structural abnormalities. Higher values indicate greater abnormality, without implying equivalent numerical scales across domains. The patterns support testing for reversible components, combined dysfunction and injury, or persistent injury; they do not establish an immune mechanism, prove irreversibility, or constitute a validated prediction model. Antibody decline alone does not establish functional recovery, and stable MRI does not exclude microscopic injury. Trajectories are illustrative, not patient data; candidate markers require reliability, appropriate controls, and multicenter validation. CSF, cerebrospinal fluid; GlyR, glycine receptor; mGluR1, metabotropic glutamate receptor 1; MRI, magnetic resonance imaging.

References

1.	Coesmans M, Smitt PA, Linden DJ, Shigemoto R, Hirano T, Yamakawa Y, et al. Mechanisms Underlying Cerebellar Motor Deficits Due to Mglur1-Autoantibodies. *Annals of neurology* (2003) 53(3):325-36. Epub 2003/02/26. doi: 10.1002/ana.10451.
